# Microwave Diagnostic Results from the Gaseous Electronics Conference RF Reference Cell

**DOI:** 10.6028/jres.100.030

**Published:** 1995

**Authors:** Lawrence J. Overzet

**Affiliations:** University of Texas at Dallas, P.O. Box 830688, EC33 Richardson, TX 75083-0688

**Keywords:** discharge, electron density, gaseous electronics, Gaseous Electronics Conference reference reactor, microwave diagnostics, microwave interferometer, plasma, temporal dependence

## Abstract

Electron density measurements with even the simplest microwave interferometry techniques can range over three to four orders of magnitude, can be responsive on time scales as fast as 50 ns, and are simple to obtain and interpret. Three groups have published electron density data taken in the Gaseous Electronics Conference (GEC) reference reactor using microwave interferometry. The agreement in the data from these groups at higher pressures is excellent especially when one considers that the GEC reactors involved have some key differences. These may have been the cause of some differences between the results obtained at low pressures, although, the manner in which the measurements were interpreted may also have contributed. The electron densities compare favorably in argon, helium, and nitrogen above 33.3 Pa (250 mTorr); but, the measurements tend to diverge some at 13.3 Pa (100 mTorr) and in 133 Pa helium above approximately 200 mA. It is speculated that the latter difference occurs as the discharges change from a bulk ionization or *α*-mode to a secondary electron emission or *γ*-mode, and that this transition occurs at lower voltages and currents for reactors with aluminum electrodes than it does for those with stainless steel electrodes. In addition, time resolved electron densities are presented. There is agreement between time resolved measurements in the two reactors, in particular, the electron density in helium discharges is found to rise dramatically after the rf excitation is turned off while the electron densities in argon and nitrogen glows exhibit only slight increases.

## 1. Introduction

A measurement of the microwave field transmission through a plasma contained in a GEC reactor can provide quick and accurate information on the spatially averaged electron concentration. This measurement can be performed using a microwave interferometer; a device which can be simple to build and operate and from which easily interpreted results may be obtained. Microwave interferometer measurements are much quicker (as fast as 20 MHz [1–4]) and less intrusive than those of Langmuir probes; but, they also are spatially averaged and usually convey no information about the electron energy distribution function. As a consequence, the two techniques are excellent complements for diagnosing the plasma state in research plasma environments.

The purpose of this article is to review the microwave interferometry data obtained from the GEC reactor to date [4–6], including a description of the measurement methods and equipment. The latter will be briefly presented in the next section on the basic equations and equipment for the sake of completeness; but, several excellent chapters [3, 7–10] and texts [11, 12] on microwave diagnostic techniques convey both more general and in depth information which is not presented here. In the final section, representative data from plasmas through argon, helium, nitrogen and carbon tetrafluoride in the GEC reactor are given as a function of the rf current and time.

## 2. Basic Equations and Equipment

**Fig. 1:**
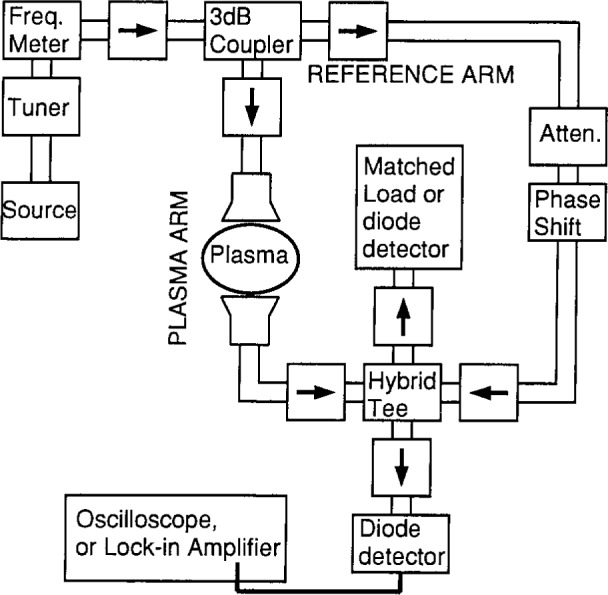
A schematic diagram of a Mach-Zehnder configuration microwave interferometer. The arrows (→) indicate isolators.

Microwave interferometers (a schematic diagram is shown in Fig. 1) allow one to measure changes in the phase length of one path with respect to another. This phase length change is measured by performing a vector addition of the microwave fields from a reference path and a path through the plasma. The resulting field is first set to a reference value with no plasma present using a precision phase changer in the reference arm of the interferometer. Often this is a null field condition (where the electric fields cancel at the detector); however, there are advantages to using the other conditions described in Sec. 4. Whatever condition is used, the result is to precisely align the electric field phasors. When the plasma is turned on, the electron density lowers the dielectric permittivity and decreases the phase length of the plasma arm. This unaligns the electric field phasors and results in a change in the electric field intensity at the diode detector. The change in the plasma arm phase length can be accurately measured by using the precision phase changer to return the system to the reference condition. The phase length change is determined by subtracting the phase angles read off the phase changer with and without plasma present. It corresponds directly to the spatially averaged electron density.

The presence of free electrons lowers the dielectric permittivity and increases the conductivity of space as follows from the Langevin equation and Ampere’s Law.

$$\varepsilon_{p}=\varepsilon_{0}\left(1-\frac{ne^{2}}{m_{e}\varepsilon_{0}\left(\nu_{m}^{2}+\omega^{2}\right)}\right)$$ (1)

$$\sigma_{p}=\frac{ne^{2}\nu_{m}}{m_{e}\left(\nu_{m}^{2}+\omega^{2}\right)}.$$ (2)

*ε*_0_ is the dielectric permittivity of free space, *ε*_p_ and *σ*_p_ are the dielectric permittivity and conductivity of the plasma, *n* is the electron concentration, *m*_e_ is the electron mass, *ν*_m_ is the electron collision frequency for momentum transfer, *e* is the electronic charge and *ω* is the microwave frequency (= 2*π f*). These equations assume that there is no static magnetic field as is the case for the GEC reactor. In the event of a static magnetic field, the permittivity and conductivity become tensors and the reader is referred to one of several more comprehensive texts [3,7,11]. The presence of positive ions in a plasma also alters the dielectric permittivity and conductivity at microwave frequencies, but, the effect is minuscule compared to that of the electrons because the ionic mass is always much larger than that of an electron.

The propagation constant of a wave in a large uniform plasma (transverse dimensions ≥5× the microwave wavelength in the plasma) is derived from the permittivity and conductivity as:

$$\gamma_{p}=\sqrt{j\omega \mu_{0}\left(\sigma_{p}+j\omega \varepsilon_{p}\right)}=\alpha_{p}+j\beta_{p}$$ (3)

where *α*_p_ is the attenuation constant and *β*_p_ is the propagation phase constant of the microwave field in the plasma. *μ*_0_ is the permeability of free space since the plasma does not greatly change this. The phase constant contains information on the plasma density and can be most easily related to the electron concentration when both the collision frequency for momentum transfer and the plasma frequency are much less than the microwave frequency. In this case, the plasma conductivity is insignificant compared to *ωε*_p_ and the phase constant becomes

$$\beta_{p}=\frac{2\pi }{\lambda_{0}}\sqrt{1-\frac{ne^{2}}{M_{e}\varepsilon_{0}\omega^{2}}}$$ (4)

where *λ*_0_ is the free space wavelength of the microwave field.

Because the plasma density in the GEC reactor can vary significantly over the one inch span between the electrodes; the average microwave propagation constant is determined from a more general formula [8].

$$\gamma_{p}+\gamma_{0}^{*}=\frac{\iint_{S}\left(\sigma_{p}+j\omega \left(\varepsilon_{p}-\varepsilon_{0}\right)\right)\;E_{p}\cdot E_{0}^{*}dxdy}{\iint_{S}\left(E_{0}^{*}\times H_{p}+E_{p}\times H_{0}^{*}\right)\cdot \hat{z}dxdy}.$$ (5)

The resulting change in the phase length of a path through the plasma is approximated by [8]

$$\Delta \phi \approx \int_{0}^{l_{p}}\left(\frac{\iint_{S}\omega \left(\varepsilon \left(x,y,z\right)-\varepsilon_{0}\right)E_{0}^{2}\;dxdy}{\iint_{S}E_{0}^{2}/\eta_{0}dxdy}\right)dz.$$ (6)

Here the microwave electric and magnetic fields are denoted by ***E*** and ***H*** with subscripts denoting plasma (p) and free space (0) conditions. The asterisks denote complex conjugates and the integration surface, *S*, is perpendicular to the beam propagation (assumed to be in the *z* direction). The length of the plasma is denoted as *l*_p_. Equation (6) applies for any dielectric perturbation such that the field intensities with the perturbation are approximately the same as those without the perturbation. (This is experimentally indicated by a constant transmitted power with and without the perturbation.) Equation (6) can be reduced further if the plasma frequency and the collision frequency are much less than the microwave frequency.

$$-\frac{2m\omega }{e^{2}}\Delta \phi \approx \frac{\int_{0}^{l_{p}}\iint_{S}n{\left|E_{0}\right|}^{2}dxdydz}{\iint_{S}{\left|E\right|}^{2}/\eta_{0}dxdy}.$$ (7)

## 3. Calibration

Equations (6) and (7) form the basis on which the microwave interferometer can be calibrated. In the published papers using microwave interferometers on the GEC reactor [4–6], the electric field intensity without plasma was measured by translating a dielectric probe in the cell and measuring the phase shift induced by that probe. Equation (6) shows how the induced phase shift is proportional to the intensity of the electric field at the position of the probe. Since the dielectric permittivity of the probe is well known, and does not depend upon position, *ε*(*x*,*y*,*z*)−*ε*_o_ is a nonzero constant only at the position of the probe. The resulting map of the induced phase shift as a function of the probe position is a direct map of the electric field intensity squared.

**Fig. 2:**
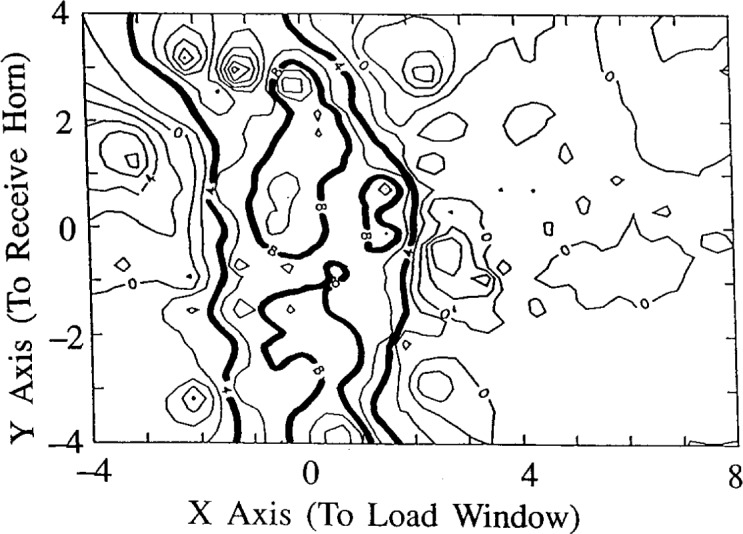
A contour plot of the phase shift obtained from translating a 7.5 mm radius nylon ball through the reactor. The 4° and 8° phase shift contours have been emboldened to indicate the beam like nature of the microwave field. (Reprinted from Ref. [6].)

Contour plots of this phase shift were published for The University of Texas at Dallas (UTD) microwave interferometer at 8.6 GHz [6] (reprinted in Fig. 2) and for the Sandia National Laboratories microwave interferometer at 80 GHz [4]. In both cases, the contours indicated the beam-like nature of the microwave field passing between the transmit and receive horn antennas. (The 4° and 8° contours in Fig. 2 have been made bold in order to emphasize the beam like nature of the microwave field.) The results do not appear as good as they might have in Fig. 2 because of the size of the dielectric probe. We used a 7.5 mm radius nylon ball in order to get easily measured phase shifts, but, it also caused some perturbation of the beam which makes the results appear poorer than they would have with a smaller probe. A smaller probe produces smaller phase shifts, however, and noise can then become a problem. Based upon these results, [6] an analytic representation for the approximate electric field intensity was formulated and used to interpret the measured phase shift. The electric field intensity of the beam was approximated by

$$E_{0}^{2}=\exp\;\left(\frac{x^{2}}{x_{0}^{2}}\right)\;\cos^{2}\left(\pi \frac{y}{y_{0}}\right).$$ (8)

Where the *y* direction goes from one electrode to the other and the *x* direction is also perpendicular to the direction of travel of the beam. The value used for *x*_0_ was 5 cm and the value used for *y*_0_ was 2.54 cm. In the *x* direction, a Gaussian shape was chosen in order to approximate the beam-like nature of the microwave field after the focusing lens. In the *y* direction, a cosine shape was chosen because of the wave guiding nature of the electrodes above and below. The electric field polarization was nominally perpendicular to the electrode surfaces, however, the cosine dependence was chosen because our measurements indicated it. The electric field approximation also assumes that the beam profile does not depend strongly upon the position along the direction of travel (the *z* axis). The beam does have some dependence along the *z* axis, however, this dependence is small. At most it introduces a 20 % programmatic error.

Once the electric field intensity is known as a function of position, Eq. (7) can be used to determine the relationship between the electron density and the measured phase shift provided the spatial distribution of the electron density is known or can be approximated. A lack of knowledge of the electron spatial distribution can be a significant source of error in unconfined geometries, particularly, since it can vary as a function of gas composition, pressure, and rf power (or current). This could result in extraordinary phenomenon in extreme cases, such as a decreasing electron density with increasing current (and power). In the GEC reactor, it is not expected that the shape of the electron density spatial distribution will change dramatically with pressure or current, and the error introduced by approximating that spatial shape is then primarily a multiplicative constant. We were able to measure the spatial distribution of electrons in the UTD reactor using a Langmuir probe in argon discharges [13] and this information was used to approximate the electron spatial distribution in Eq. (7) for all the results presented here. The electron concentration has a cylindrical symmetry unlike that of the microwave field. We have chosen to approximate this dependency as

$$\begin{array}{ll} n_{e}\left(r\right)=n_{e0} & r\leq r_{e}\\ \;=n_{e0}\;e^{-\left(r-r_{e}\right)/L} & r_{e}\leq r\leq 12.5\;\mathrm{cm}. \end{array}$$ (9)

Where *r*_e_ is the radius of the electrodes, the reactor wall is at 12.5 cm and *r* = 0 is on the reactor axis. The value for *L* was set to 3 cm [13]. The profiles for the electric field and the electron concentration were then placed into Eq. (7) and the three dimensional integration yielded a value for the electron density *n*_e0_ between the electrodes of the cell of 1.3×10^9^ electrons cm^−3^ per degree of phase shift. We estimate that this number is accurate to within ±30 % based upon an estimated ±20 % error in measuring and approximating the electric field intensity and a 10 % error in approximating the electron spatial distribution. The precision and repeatability of the results is much better, approximately the greater of ± 0.2° or 3 %. It should be noted that the expansion of the electrode sheaths with decreasing pressure is not accounted for in this calibration. This correction could be important for very low pressure discharges, particularly in helium where the sheath thicknesses can be large; but, is generally less than 10 % for the sheath thicknesses encountered in other gases.

## 4. Equipment Issues Peculiar to the GEC Cell

Most of the electron density data have been obtained using the Mach-Zehnder configuration and the simplest detection techniques as shown in Fig. 1 [4, 6]. This instrument is often called the “bridge” interferometer, and it has several advantages over the more complex heterodyne, serrodyne and frequency modulated interferometers [3]. Chief among these advantages for operation in the GEC reactor is their simplicity of design, economy, and speed. The speed in following electron density changes is limited only by the response of the detector in the case of the bridge interferometer and can be as large as 20 MHz [1–4]. In addition, phase changes of considerably less than 0.1° can be measured by using a lock-in amplifier. The chief disadvantage is the ambiguity in phase shifts above 180° or 360°; however, this situation is rarely encountered in this experimental research cell. The opposite problem, phase shifts less than 1° is more typically encountered for electronegative plasmas.

**Fig. 3:**
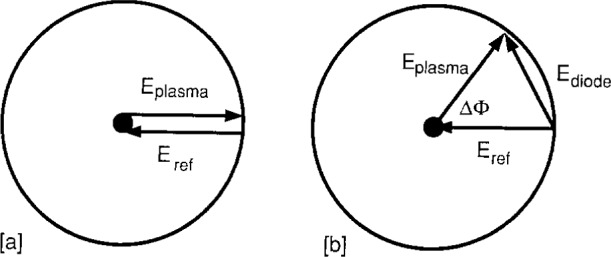
Phasor diagrams of the electric fields at the diode detector (*E*_diode_) due to those from the plasma (*E*_plasma_) and reference arm (*E*_ref_) of the microwave interferometer. a) A nulled field condition showing how the equal amplitude fields at opposite phase cancel. b) The value of *E*_diode_ caused by a phase change of Δ*Φ*.

Both the Sandia [4] and UTD [6] research groups have used detection techniques other than the null field approach in order to improve the sensitivity to small phase changes. A pair of diode detectors is used on the *E* and *H* plane arms of a matched hybrid tee on the UTD interferometer; while the Sandia group used a 90° phase shift [14] after the null field condition was found. The reasons behind using these phase detection techniques are most readily explained by examining how the vector fields add (or subtract) at the detector(s) using a phasor diagram. The field phasors are shown in Fig. 3 (a) for a null field condition (with no plasma) at a single detector and for a plasma condition causing a Δ*Φ*° phase shift in Fig. 3 (b). The electric field intensity at a single diode for the null field condition is zero, and the resultant voltage on the diode is also zero. When the plasma causes the phase length of the plasma arm to decrease by Δ*Φ*, the fields no longer cancel and the field at the diode detector becomes *E*_diode_= 2*E*_ref_sin(Δ*Φ*/2). (This assumes there is no attenuation of the signal through the plasma arm and that the power transmitted through the plasma and reference arms are the same. The exact form of the equations would change some if these conditions did not hold, but, the essential results would be the same.) The voltage on the diode detector is just the square of *E*_diode_ for a square law detector so that *V*_diode_ becomes proportional to sin^2^(Δ*Φ*/2). Since sin(Δ*Φ*/2) is approximately equal to Δ*Φ*/2 at small phase angles, the diode signal rises as (Δ*Φ*)^2^ and the sensitivity of the instrument is very low.

**Fig. 4:**
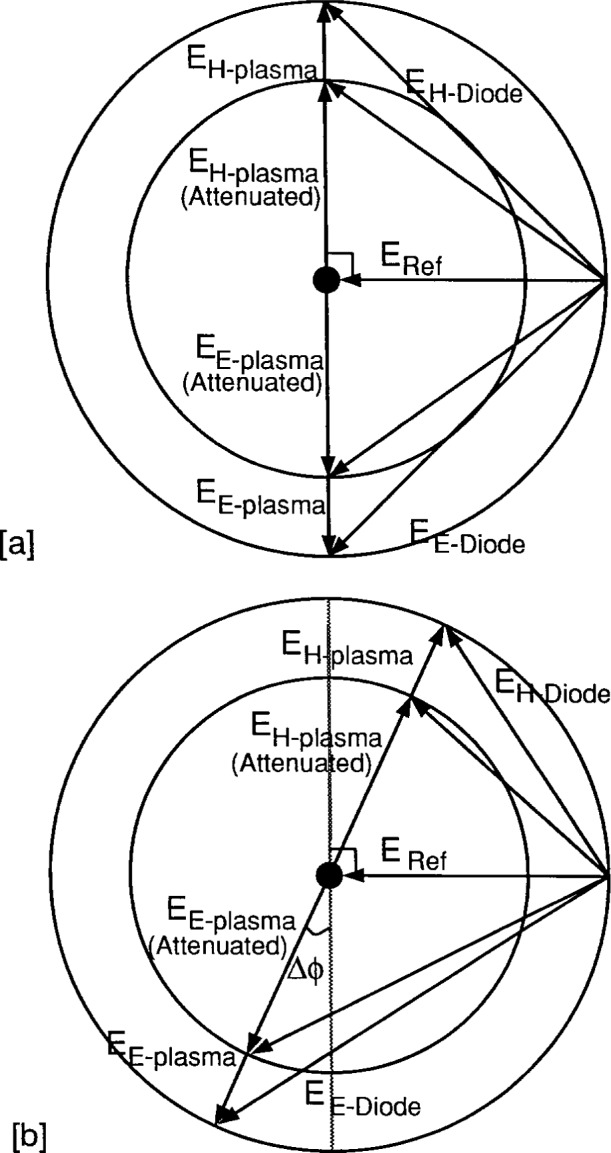
Phasor diagrams of the electric fields at the *E*-plane and *H*-plane diode detectors (*E_E_*_-Diode_ and *E_H_*_-Diode_) of the Hybrid Tee due to those from the plasma (*E_E_*_-plasma_ and *E_H_*_-plasma_) and reference arm (*E*_ref_) of the microwave interferometer. The plasma and reference arm electric fields add going into the *H*-plane arm of the Hybrid Tee, but subtract going into the *E*-plane arm. a) A nulled field condition showing how either equal amplitude or unequal amplitude electric fields in the reference and plasma arms cause equal *E*-plane and *H*-plane diode signals. b) The value of *E_E_*_-Diode_ becomes greater than *E_H_*_-Diode_ by a phase change of −Δ*Φ*.

The change in the diode voltage becomes approximately proportional to Δ*Φ* if one offsets by 90° from the null field condition because sin^2^((Δ*Φ* + 90°)/2) is equal to 1/2(1−sin(Δ*Φ*)). The signal is now proportional to Δ*Φ*, but, rides on a significant dc offset voltage. This is the offset condition used by the Sandia group [14] and it is the most sensitive detection position using a single diode detector. The matched pair of diodes technique used at UTD also makes the diode voltage proportional to the phase shift, but, does so at a null voltage condition. A phasor diagram of the fields at the diode detectors for this technique is shown in Fig. 4. Now the outputs of the two diode detectors are set to be equal by placing the field from the plasma arm 90° offset from that in the reference arm. The difference in the voltage between the two diodes on the *E* and *H* plane arms of the hybrid tee is then measured using a differential amplifier. The phase shift induced by the plasma causes the plasma arm fields to rotate in the phasor diagram and results in a decrease in *E*_*H*-diode_ and an increase in *E*_*E*-diode_ in Fig. 4 (b). The difference in the signals can be found by assuming square law detectors and small phase shifts to be:

$$V_{H–\text{diode}}-V_{E–\text{diode}}\approx 4E_{\text{Ref}}\;E_{p}\;\Delta \Phi .$$ (10)

This technique allows one to determine the phase shift using a null voltage condition without having to vary the setting of the precision attenuator in the reference arm. This can be seen in Fig. 4 (a) where the inner circle represents an attenuated plasma arm field. The phase shifter can still rotate the fields into a null voltage condition. This feature can be both beneficial and detrimental. Some precision attenuators can cause a variation in the phase length as the attenuation is increased since they add a leaky dielectric to the waveguide. This detection technique allows one to avoid the programmatic errors that these attenuators would introduce. It also allows one to send the maximum power available to the diodes through the reference arm rather than requiring that the plasma and reference arm power be equalized and this can increase the signal output for a given phase length change. It does not give one information on how much the beam is being attenuated by the plasma as a matter of course and this information can be necessary for some circumstances.

## 5. Results

**Fig. 5:**
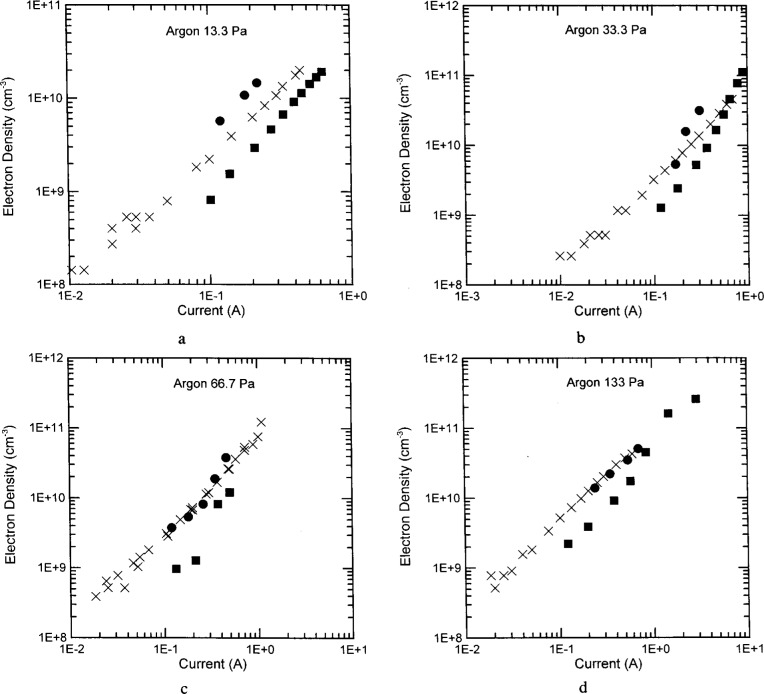
The electron densities of argon discharges as a function of the discharge current amplitude at the fundamental driving frequency. The ×’s are data from the UTD reactor. The filled dots are data from the Sandia National Laboratories reactor [4]. The filled squares are data from the Wright Patterson Air Force Base reactor [5]. (a) 13.3 Pa. (b) 33.3 Pa. (c) 66.7 Pa. (d) 133 Pa.

The electron densities in Ar (Fig. 5), He, N_2_ and CF_4_ plasmas are all plotted as a function of the discharge current at the fundamental rather than the voltage for two reasons. First, the peculiarities of the UTD cell did not allow both voltage magnitude and phase angle between current and voltage to be in agreement with the standard cells at the same time [6]. It was decided that since the phase angle between current and voltage was always in agreement with the standard as a function of current (but not always as a function of voltage) that current should be used as the independent variable. Second, the current density can be related to the electron density through the simplistic formula *J* = *nev* applying to the body of the glow and implying that the electron density and current should have a simple relationship. This is born out somewhat in Fig. 5. The electron density measured in the UTD reactor is always linear on this log-log scale; and in fact, all the electron density traces nearly sit on a single power law line independent of pressure. The relationship between electron density and voltage amplitude is not so simplistic since the discharge impedance can be a complicated function of the glow parameters and in particular the powered electrode sheath. The electron density has been plotted versus the total current rather than the current density in these figures because the actual relationship between the total discharge current and the current density in these unconfined plasmas is not precisely known.

The electron density results at different pressures and from different laboratories have been plotted for comparison in Fig. 5. The ×’s are data obtained from the UTD reactor, the filled dots are from the Sandia National Laboratory (SNL) reactor [4] and the filled squares are from the Wright Patterson Air Force Base reactor (WPAFB) [5]. Several facets of the data in Fig. 5 are appropriate for discussion. First, the data from both the SNL and WPAFB reactors have been normalized for the extension of the electron density beyond the electrode ring in order to make all three sets compatible. This was not incorporated in the publication of these data, and amounts to a reduction in those reported electron density values by a factor of 1.6. Even with this reduction, it is clear that the electron density measured in the SNL reactor at 13.3 Pa was significantly larger than that measured in either of the other two reactors. In fact, the differences between the SNL and WPAFB data sets is nearly a factor of 4 with the UTD results splitting the difference. At higher pressures, the agreement between the SNL and UTD reactor results is excellent while the electron densities of the WPAFB cell are generally somewhat smaller.

The reasons for the differences at 13.3 Pa are not nearly as clear as one could wish because there are too many possible explanations. Arguably the best explanation is that the electron densities are just not equal in these reactors at a given current because of the physical and electrical differences between them. While both the SNL and WPAFB cells have aluminum electrodes, the UTD cell has steel electrodes with the shower-head electrode powered and a pinhole welded into the grounded electrode. These differences are substantial enough to cause a 30 % reduction in the discharge current for a given electrode voltage [6]. It may also influence the electron density of the plasma, particularly at low pressures. In addition, the SNL cell was driven symmetrically while the WPAFB and UTD reactors were driven asymmetrically. Unfortunately, the effect this should have on the electron concentration of the plasma is not particularly clear. Another potentially important difference is the frequency of the microwave interferometers involved. The SNL interferometer was at 80 GHz and had a beam width of approximately 8 mm [4]. This beam could be centered between the electrodes very accurately, and sampled the plasma only where the electron density was large. The WPAFB (35 GHz) and UTD (8.6 GHz) interferometers both effectively filled the entire volume between the upper and lower electrodes. Thus, these interferometers measured an electron density which was averaged over the sheath regions as well as the center of the glow. This would cause the reported electron density values to be lower by approximately a factor corresponding to the fraction of the volume between the electrodes taken up by the sheaths. This effect would be larger at lower pressures since the sheath expands; and it fits the trend in the data of better agreement at higher pressures. Unfortunately, the sheaths are not large enough to account for the difference between the SNL and WPAFB results at 13.3 Pa in argon.

**Fig. 6:**
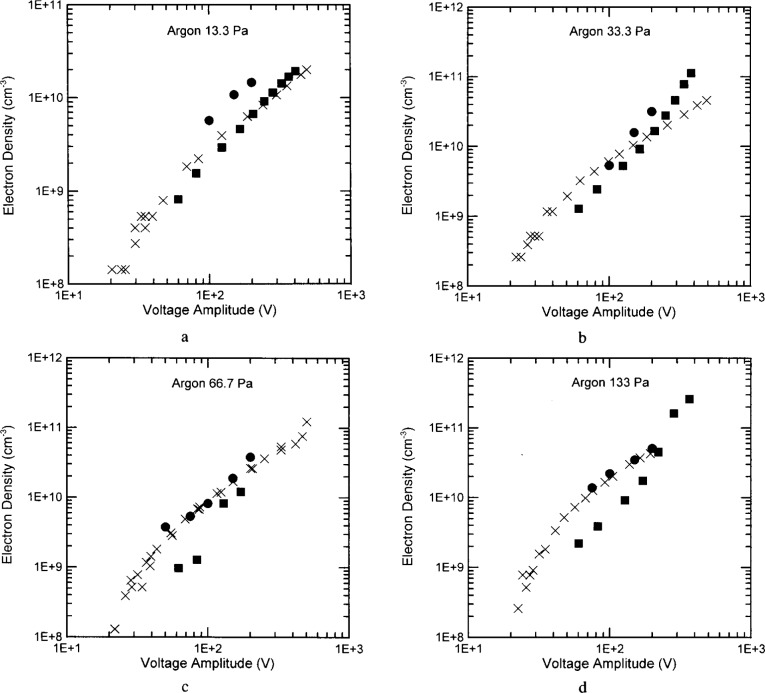
The electron densities of argon discharges as a function of the electrode voltage amplitude at the fundamental driving frequency. The symbols are as in Fig. 5. (a) 13.3 Pa. (b) 33.3 Pa. (c) 66.7 Pa. (d) 133 Pa.

A final possibility is that current is not a good independent variable. This is illustrated in Fig. 6 where the electron density has been plotted for Ar at 13.3 Pa as a function of the electrode voltage. The agreement between the UTD and WPAFB results is excellent here. (This indicates that the *I–V* curves between these cells are significantly different.) Accounting for the fraction of the plasma volume taken up by sheaths will even increase the agreement between these results. Interestingly, the plots at other pressures are not affected nearly as much so the results agree closely there as well. There are two possible reasons why the plasma current may not be the best independent variable. First, the plasma current must be calculated by accounting for the equivalent circuit between the *I-V* probes and the electrode surface. This is made easier using the shunt circuit [15], but still involves a more careful procedure than the voltage measurements as well as more potential for experimental error. Second, the plasma current is determined more by the sheath capacitance (and the sheath voltage) at these low pressures than by the electrons in the body of the glow. As a consequence, the current density in the body of the glow could be determined much more by the average electron velocity than by the density and subtle changes in reactor geometry can lead to significant differences in the measured *I–V* curves.

**Fig. 7:**
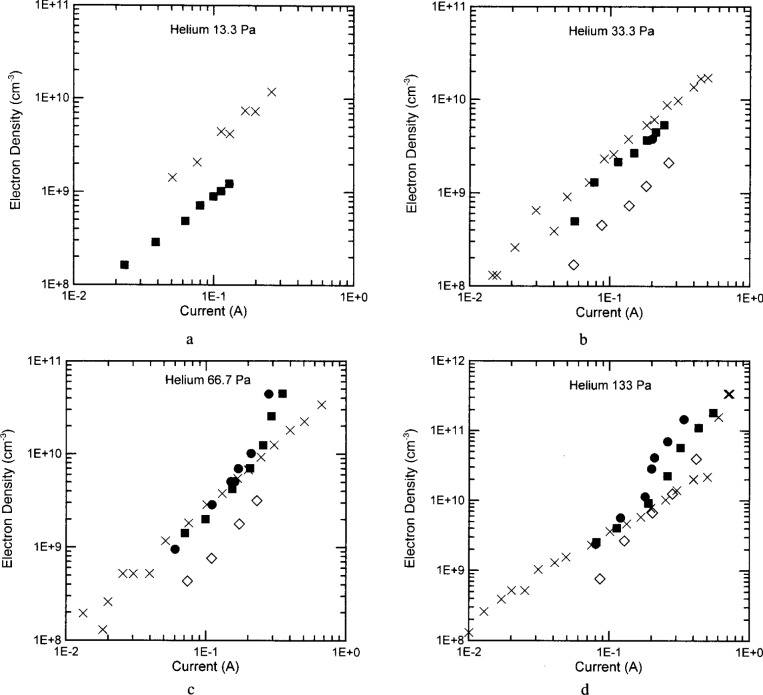
The electron densities of helium discharges as a function of the discharge current amplitude at the fundamental driving frequency. The ×’s are data from the UTD reactor. The filled dots are data from the Sandia National Laboratories reactor [4]. The filled squares are data from the Wright Patterson Air Force Base reactor [5]. The open diamonds are from laser induced fluorescence measurements in the National Institute of Standards and Technology reactor [6]. (a) 13.3 Pa. (b) 33.3 Pa. (c) 66.7 Pa. (d) 133 Pa.

The electron densities of helium discharges are plotted in Fig. 7. The symbols are as in Fig. 5, except that the open diamonds are from laser induced fluorescence (LIF) measurements made at The National Institute of Standards and Technology [16]. The electron density of helium discharges increased with increasing rf current at all pressures just as in argon. In addition, the electron concentrations of both argon and helium discharges (in the UTD reactor) sit on nearly the same power law line independent of the pressure. (*N*_e_ = 7.1×10^10^ *I*^4/3^.) The similarity in the electron concentrations as a function of the rf current is surprising since the electron-neutral collision frequency in helium and argon have significantly different energy dependencies as well as magnitudes [17], the electron loss rates due to ambipolar diffusion can be significantly different (because of the ion masses and mobilities) [4, 18] and the sheath width in helium is generally significantly larger than in comparable argon discharges [19]. These differences cause the electron energy distribution functions of these two gases to be quite different as well [20, 21]. The EEDF in helium is much more Maxwellian, while the EEDF in argon is better characterized by a Druyvesteyn energy distribution. Apparently, these effects do not cause significant differences in the electron concentrations of helium and argon discharges as a function of the discharge current; even though they do cause the rf voltage of helium discharges to be much larger than that of comparable argon discharges [19].

**Fig. 8:**
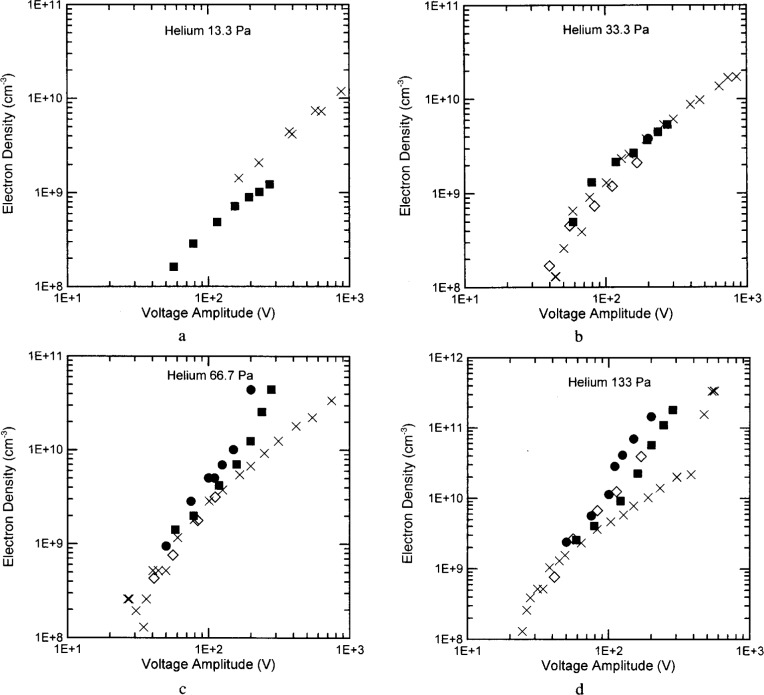
The electron densities of helium discharges as a function of the electrode voltage amplitude at the fundamental driving frequency. The symbols are as in Fig. 7. (a) 13.3 Pa. (b) 33.3 Pa. (c) 66.7 Pa. (d) 133 Pa.

The electron densities in helium exhibit significant differences at low pressures again, even though there is substantial agreement at higher pressures. The differences between the electron densities of the WPAFB and UTD reactors are minimized when the electron density is plotted as a function of the electrode voltage rather than the plasma current again. (See Fig. 8.) The electron density in the SNL and WPAFB reactors increases abruptly for both the 66.7 Pa and 133 Pa discharges around 0.2 A to 0.3 A while the electron density in the UTD reactor continued a linear increase (on the log-log plot.) The UTD electron densities in the 133 Pa discharge may exhibit a similar abrupt increase at a larger current (near 0.7 A). A possible explanation for this difference is related to the transition from the *α*-mode to the *γ*-mode of the discharge [4,20,22]. Godyak, Piejak and Alexandrovich have indicated that the electron concentration rapidly rises and the electron temperature rapidly decreases as the discharge current (and voltage and power) are moved through this transition from a local to a nonlocal primary ionization source [20]. The *γ*-mode discharge is sustained by an electron avalanche in the powered electrode sheath in much the same manner as the cathode glow is sustained in a dc discharge. The electron avalanche occurs when the sheath voltage is large enough to produce significant levels of secondary electron emission from the electrode as well as electron ionization in the sheath and glow edge. The secondary electron emission coefficient of energetic helium ions is generally larger than that of argon ions [23] and should also be larger on the aluminum electrodes of the SNL and WPAFB reactors than on the stainless steel electrodes of the UTD reactor. This would cause those reactors to transition into the *γ*-mode at lower currents and voltages than the UTD reactor and consequently to have a larger electron concentration above the transition point. It could also explain why the argon discharges do not exhibit the same kind of abrupt increase; since, the secondary electron yield is expected to be smaller for argon ions in addition to the already smaller rf voltages typical of those discharges.

The Laser Induced Fluorescence (LIF) measurements of the electron density [16] sit below the microwave interferometry results when plotted as a function of the rf current, but, are in excellent agreement with the microwave results when plotted as a function of the rf voltage. This again indicates a discrepancy between the *I–V* characteristics of these cells; however, the close agreement of these results versus the rf voltage may also just be fortuitous. The LIF results have been interpreted using the excitation coefficients of Sobelman and coauthors [24]. These excitation coefficients are thought to be the best available, but, are about a factor of 6 lower than those of Griem [25]. Using Griems’ coefficients gives results that agree closely with the microwave interferometer measurements when plotted versus the rf current, but not versus the rf voltage! As a consequence, it is not clear which is actually better at this point.

**Fig. 9:**
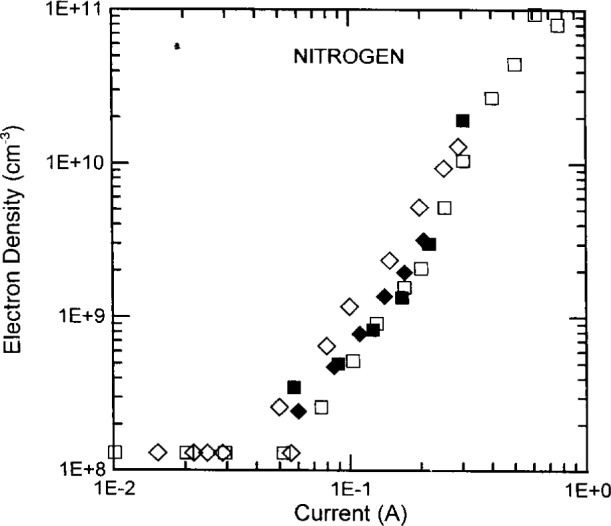
The electron densities of nitrogen discharges. Open symbols are data from the UTD reactor at 13.3 Pa (diamonds) and 40 Pa (squares). Filled symbols are data from the WPAFB reactor at 13.3 (diamonds) and 33.3 (squares).

**Fig. 10:**
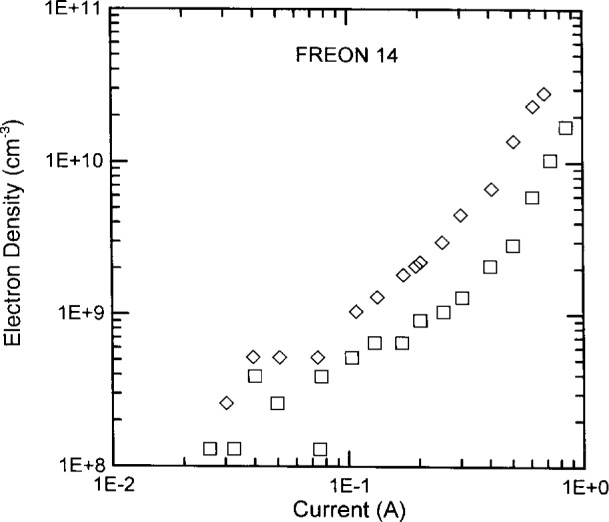
The electron densities of carbon tetrafluoride discharges in the UTD reactor. Open diamonds represent data at 13.3 Pa, open squares at 40 Pa.

The electron densities of nitrogen and CF_4_ discharges at 13.3 Pa and 40 Pa in the UTD reactor are plotted in Figs. 9 and 10 as open diamonds and squares. The electron densities in nitrogen at 13.3 Pa and 33.3 Pa in the WPAFB reactor are plotted as filled diamonds and squares in Fig. 9. The lowest electron density data points in both these figures correspond to 0.1° of phase shift (1.3×10^8^ cm^−3^). 0.1° is the smallest phase shift which can be resolved on the UTD phase changer without using any enhanced detection techniques. Unfortunately, the electron densities of nitrogen discharges below 60 mA can be significantly smaller than 1.3×10^8^ cm^−3^. As a consequence, we did not measure the electron concentrations there, and, the data points at those currents only indicate an upper limit for the electron density.

While the UTD reactor data for the noble gases indicated an electron density which increased slightly with increased pressure or had little pressure dependence; the data in both nitrogen and CF_4_ indicate an electron concentration which decreased with increased pressure. An increased electron attachment rate with increased pressure is the most likely reason in the case of CF_4_, but, the reason for this phenomenon in the case of nitrogen is not as clear. It may be caused by a transition from a “sheath oscillation” electron heating mechanism toward an ohmic heating mechanism in the bulk of the discharge as noted by Turner for nitrogen [26] and Godyak, Piejak and Alexandrovich for argon discharges [20]. Finally, unlike argon, helium and nitrogen, the CF_4_ electron densities are nonlinear on the log-log scale most likely because the composition of the plasma changes with increasing current (and voltage and power.)

**Fig. 11:**
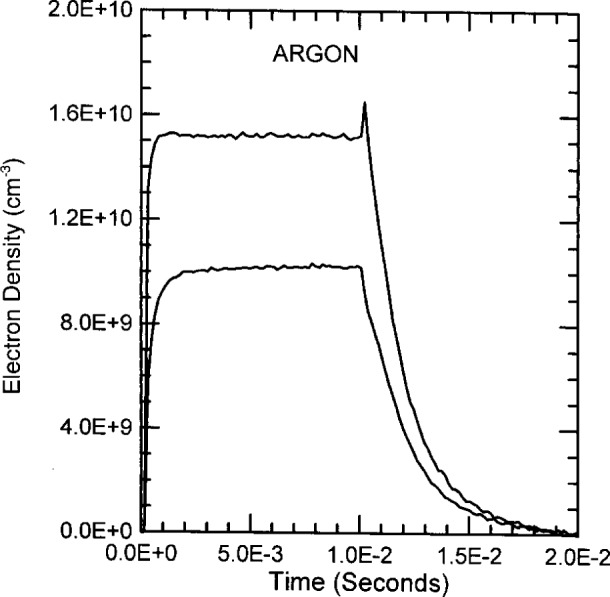
The time dependence of the electron density in pulsed argon discharges at 33.3 Pa. Lower curve: 0.25 A, 170 V, *V*_dc_ = − 77 V. Upper curve: 0.46 A, 350 V, *V*_dc_ = − 168 V. All values are amplitudes taken at the approximate steady state condition.

**Fig. 12:**
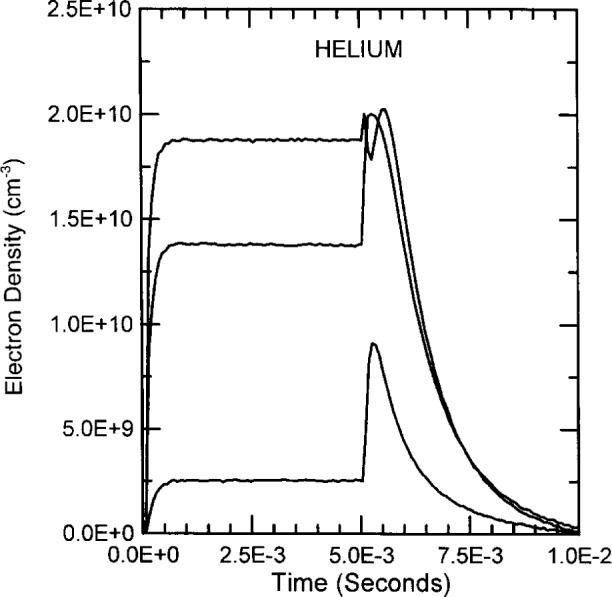
The time dependence of the electron density in pulsed helium discharges at 40 Pa. Lowest curve: 0.093 A, 170 V, *V*_dc_ = − 98 V. Center curve: 0.25 A, 505 V, *V*_dc_ = − 107 V. Upper curve: 0.34 A, 740 V, *V*_dc_ = − 108 V. All values are amplitudes taken at the approximate steady state condition.

**Fig. 13:**
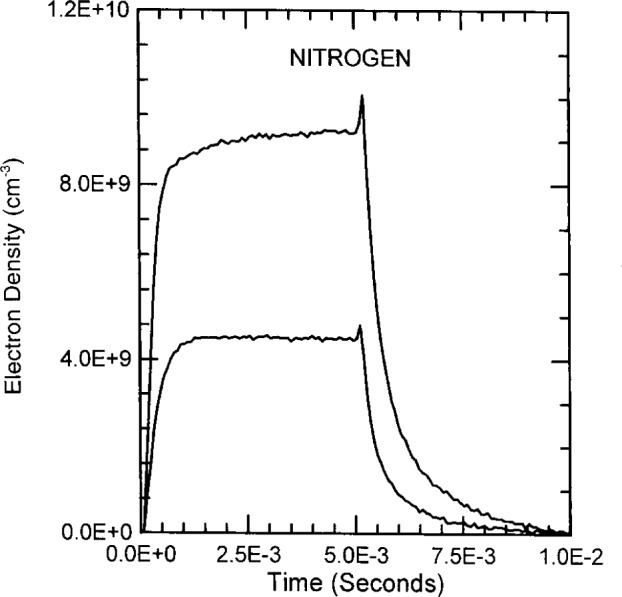
The time dependence of the electron density in pulsed nitrogen discharges at 33.3 Pa. Lower curve: 0.18 A, 280 V, *V*_dc_ = − 87 V. Upper curve: 0.27 A, 360 V, *V*_dc_ = − 99 V. All values are amplitudes taken at the approximate steady state condition.

The time dependent electron densities of pulsed discharges through argon, helium and nitrogen are plotted in Figs. 11, 12 and 13. Argon discharges exemplify ordinary behavior. The electron density rises during the first 1 ms to 2 ms to a steady state value. When the rf is extinguished at 10 ms, the electron concentration decreases exponentially in time with a decay time of approximately 2.5 ms which holds to the lower end of the microwave interferometer range. This decay time is the same for both the 0.25 A and 0.46 A discharges. There is a slight increase in the electron concentration just after the rf excitation is turned off for the larger current. This small increase may be caused by argon atoms in a metastable excited state and will be discussed more in conjunction with the helium results next.

The electron densities in helium discharges reach steady state within approximately 0.5 ms to 1 ms. The electron concentration increases significantly immediately after the rf excitation is turned off as was first described by Greenberg and Hebner for the GEC reactor [4] and by Biondi [27] for microwave excited discharges. The amount of the increase appears to be substantially independent of the initial electron concentration and the subsequent decay in the electron concentration is exponential with approximately a 1.2 ms time constant. The shorter decay time compared to argon is caused by the lighter ion mass and higher ion mobility [4, 18]. The initial increase in the electron concentration at rf turn off is thought to be caused by a rapidly decreasing electron loss rate in the afterglow (caused by electron cooling) coupled with an ionization rate that does not decrease quickly because of the presence of helium atoms in the 2 ^3^S and 2 ^1^S metastable electronic states. These excited state atoms can produce ionization through metastable-metastable collisions as well as superelastic electron collisions followed by electron impact ionization of a metastable state atom.

The electron concentration in nitrogen rises quickly to steady state at the low current, but, does not reach a good steady state within 5 ms for the discharge at 0.27 A. The electron concentration increases a small amount after the rf excitation is extinguished before decreasing in the afterglow. While the electron density decay in nitrogen is faster than in either argon or helium, it is not exponential in time. The increased loss rate for electrons in nitrogen compared to argon and helium indicates an additional loss mechanism. The most likely mechanism is electron ion gas phase recombination. This is an inefficient mechanism in low pressure atomic gases, but, can be significantly more efficient in molecular gases where energy and momentum can be more easily conserved.

## 6. Conclusion

The electron densities of discharges through argon measured by microwave interferometry in different GEC reactors show substantial agreement at pressures above 13.3 Pa. At 13.3 Pa, there were some substantial differences in the measurements which may have been caused by differences in the reactors or the interferometers themselves. It is not clear whether one or the other or even both may be most important. The electron densities of discharges through helium show substantial agreement at low currents; but, are dissimilar at large currents. The reason for the discrepancy at large currents is postulated to be the mechanism by which the discharge is sustained. It appears that glow discharges formed using aluminum electrodes transition into *γ*—mode at lower voltages and currents than do discharges formed between stainless steel electrodes.
